# Saliva NIR spectroscopy and Aquaphotomics: a novel diagnostic approach to Paratuberculosis in dairy cattle

**DOI:** 10.3389/fcimb.2024.1395949

**Published:** 2024-11-13

**Authors:** Saba Behdad, Abbas Pakdel, Reza Massudi

**Affiliations:** ^1^ Department of Animal Science, College of Agriculture, Science and Research Branch, Islamic Azad University, Tehran, Iran; ^2^ Department of Animal Science, College of Agriculture, Isfahan University of Technology, Isfahan, Iran; ^3^ Laser and Plasma Research Institute, Shahid Beheshti University, Tehran, Iran

**Keywords:** Aquaphotomics, Johne’s disease, *Mycobacterium avium subspecies Paratuberculosis*, Near-Infrared spectroscopy, saliva

## Abstract

**Introduction:**

Paratuberculosis is a granulomatous intestinal infection that affects ruminant animals worldwide. The disease is often detected when most animals are already infected due to the long incubation period and the high transmissibility of the infectious agent. The lack of a comprehensive method to diagnose Paratuberculosis is a global challenge. Therefore, a non-destructive, fast, and cost-effective diagnostic method for early detection of Paratuberculosis is crucial.

**Methods:**

Near-infrared spectroscopy (NIRS) and Aquaphotomics have the potential to diagnose the disease by detecting changes in biological fluids. This study aimed to investigate the diagnostic ability of NIRS and Aquaphotomics for Paratuberculosis in dairy cattle by monitoring and data mining of saliva. The diagnostic models were developed according to saliva spectra of dairy cattle in the NIR range and 12 water absorbance bands from 100 to 200 days after calving in two groups: positive and negative, based on the same results of seven ELISA tests of blood plasma, as a reference test.

**Results:**

Both NIRS and Aquaphotomics methods had high diagnostic accuracy. Using QDA and SVM models, 99% total accuracy, 98% sensitivity, and 100% specificity were achieved in internal validation. The total accuracy in external validation was 90%. This study presents two novel approaches to diagnosing Paratuberculosis in dairy cattle using saliva.

**Discussion:**

The study found that changes in water absorbance spectral patterns of saliva caused by complex physiological changes, such as the amount of antibody related to Paratuberculosis in dairy cattle as biomarkers, are crucial in detecting Paratuberculosis using NIRS and Aquaphotomics.

## 1 Introduction

Paratuberculosis, also known as Johne’s disease, is a bacterial infection caused by *Mycobacterium avium* subspecies *Paratuberculosis* (MAP). This type of bacteria is acid-fast and Gram-positive, and it often leads to disease outbreaks in ruminants, affecting the entire herd (OIE Terrestrial Manual 2021- PARATUBERCULOSIS (JOHNE’S DISEASE) 2021.pdf, 2021; Pickrodt et al., 2023). Johne’s disease can be transmitted through horizontal and vertical transmission. In horizontal transmission, the bacteria is mainly transmitted through fecal contamination of the udder or pasture, water, food, colostrum, and sometimes through aerosol (Lee et al., 2023). In vertical transmission, MAP is transmitted from the infected dam to the embryo through the placenta. Although the infection starts locally, it can turn into a systemic pattern and cause chronic granulomatous enteritis, which can lead to animal death (Marquetoux et al., 2019; Correa-Valencia et al., 2021).

Paratuberculosis has both direct and indirect economic costs. The direct economic effects of the disease include reduced growth rate, lower meat and milk production, premature culling of dairy cows, higher mortality, and increased costs due to compensation. The invisible effects include reduced fertility or infertility, disease control costs, diagnostic test costs, abortions, infected calves born, susceptibility to other diseases, and veterinary costs. The indirect economic impacts of this disease include the cost of disease control, revenue foregone due to restricted market access, export losses, losses to other sectors in the supply chain and consumers, impact on animal health and welfare, marketing, and public health-related issues, productivity reduction, loss of business and market, decrease in market value, and food insecurity (Barratt et al., 2019). Johne’s disease has a long incubation period; as a result, it can remain hidden in the herd for a long time, and for this reason, it is classified into four stages: silent, subclinical, clinical, and advanced (Hussain et al., 2016). Calves can be affected from the embryonic period to the first months of birth, although the clinical symptoms may not be revealed for years. During this period, other animals can be exposed to contamination through feces, environment, food, and milk of infected animals (Fecteau, 2018).

Infection prevention and control systems are essential for preventing the spread of diseases. Unfortunately, identifying infections in a herd often occurs after the bacterial strain has already spread (Karuppusamy et al., 2021). Therefore, it is essential to promptly identify and isolate infected animals, and vaccinate the herd to control the spread of the infection. It is crucial to note that introducing infected animals into a herd can significantly increase the risk of bacterial transmission (Chaubey et al., 2016). Although Johne’s disease has a vaccine, it is not widely available in many countries and does not provide complete immunity. In addition, there are concerns about using this vaccine because it is difficult to diagnose tuberculosis in dairy cattle. Therefore, it is crucial to prevent the disease at the farm level and reduce its transmission from one generation of dairy cattle to another. Currently, *Mycobacterium avium* subspecies *Paratuberculosis* (MAP) continues to be prevalent, especially in dairy cattle breeding systems, due to factors such as the long-term pre-clinical shedding stage, poor management practices, suboptimal laboratory tests, and the environmental persistence of the bacteria (Garvey, 2020). It is crucial to use appropriate diagnostic tests and approaches for the rapid identification of MAP infection and its spread (McGregor et al., 2015). The primary diagnostic tests for MAP infection are to identify the strains of bacteria and the host’s immune response to the bacteria infection. Two techniques commonly used to diagnose Paratuberculosis strains are bacterial culture and polymerase chain reaction (PCR) tracking of molecular components (Assessment of surveillance and control of Johne’s disease in farm animals in GB, 2002).

The enzyme-linked immunosorbent assay (ELISA), complement fixation test (CFT), and agar gel immunodiffusion (AGID) are commonly used molecular techniques to evaluate the immune response of the host (Sardaro et al., 2017). However, the accuracy and sensitivity of each test are different. Therefore, in the advanced stages of the disease, it is recommended to use a set of tests. However, the choice of tests can be influenced by factors such as cost and logistics (Rieger et al., 2021). It is important to note that the molecular tests mentioned are highly effective in early screening and tracking, and histopathological studies provide a definitive and accurate diagnosis of the disease. ELISA is the most cost-effective tool, but the PCR or FC test is preferred to reduce prevalence, and both are considered more sensitive for low-shedding animals (Robins et al., 2015; Smith et al., 2017).

Detecting infection in primary samples using the right diagnostic test is the first step in controlling the spread of infection. This provides rapid identification of infected animals, which is of great economic importance in predicting the disease state (Chaubey et al., 2016). Several diagnostic tests are available, and their accuracy, sensitivity, and cost-effectiveness must be considered. Some experts believe using ELISA and PCR methods together would be most cost-effective (Aly et al., 2012). However, it is worth noting that the lack of a non-invasive, accurate, fast, and available diagnostic method for Johne’s disease, in each of the four stages, affects the economic importance of infection control.

Because of the lack of early and accurate diagnostic tests and the inherent resistance of MAP to antibiotics and disinfectants, controlling infections has become extremely challenging. This has turned Paratuberculosis into a global issue. Therefore, there is an urgent need to develop an accurate diagnostic method that can distinguish between healthy and infected animals based on the disease agent that produces antibodies. This method should also show the animal’s condition at each stage of the disease. Nowadays, modern physical methods, such as lasers and spectroscopy, are being used to diagnose and treat various diseases in humans, plants, and farm animals. This is due to their fast, accurate, non-invasive, portable, and minor side effects.

Near-infrared (NIR) spectroscopy is a non-invasive and physical method that has been increasingly applied for the last 50 years. This technique does not require any sample preparation or chemical pollution and it is based on measuring molecular bond vibration. It is widely used to assess the quality of agricultural and pharmaceutical products by studying the interaction of electromagnetic waves with biological materials. Recently, NIR spectroscopy has been applied to the structural analysis of water, making it an even more valuable tool (Munćan et al., 2019).

NIR spectroscopy is a method that can estimate multiple components in a sample at once. To do this, calibration equations are developed. In most cases, the reproducibility of sample analysis is equal to or better than that of chemical methods. Additionally, since this method requires small sample sizes and is non-destructive, the sample can be recovered and used for other purposes. A multivariate analysis model, NIR spectroscopy can predict the chemical composition of unknown samples. This model describes the relationship between the NIR absorption (or transmittance) spectrum and the chemical constituents of interest, using Bir-Lambert’s law. This allows the composition of unknown samples from the same population to be predicted, named chemometric (Tsenkova et al., 2018). Other advantages of using the NIR spectroscopy method are continuous manitoring during milking. Recent research on diagnosing Johne’s disease has explored modern physical methods like monitoring gene expression patterns in salivary glands (Sanjay Mallikarjunappa et al., 2019) and other methods (Le Puil et al., 2006; Norby et al., 2006; Yakes et al., 2008; Roussel, 2011; Smith, 2016; Tooloei et al., 2016; Mathie, 2017; Agrawal et al., 2020; Karuppusamy et al., 2021). However, despite these studies, developing a non-invasive, accurate, fast, available, and cost-effective method to diagnose all four stages of Johne’s disease, particularly in its early stages, remains a global challenge.

Hydrogen bonds in water can cause issues in infrared (IR) spectroscopy due to high absorbance and interference with the absorption of other components. NIR spectroscopy is preferred as it reduces these problems. In 2005, a new approach called Aquaphotomics was developed. It examines water absorbance bands and their changes as a biomarker, using water memory and mirroring principles (Tsenkova et al., 2018). This approach provides a unique opportunity to describe the complex state of water using its multidimensional NIR spectra (Munćan et al., 2019). Over the years, studies focused on water structures and proving the memory and mirror-like principles of water have been widely conducted in various fields, for example, diagnosis of diabetes (Li et al., 2020) and Alzheimer’s disease in humans (Tsenkova, 2007), diagnosis of pneumonia (Santos-Rivera et al., 2021) and mastitis (Tsenkova, 2007), the detection of estrus in dairy cattle (Kinoshita et al., 2015; Unalan, 2016; Iweka et al., 2020), the detection of adulteration in powdered milk (Santos et al., 2013) and honey (Yang et al., 2020), the detection of quality and freshness of meat (Mileusnić et al., 2017), and the investigation of quantitative and qualitative traits of transgenic organisms (Sohn et al., 2021).

The Water Matrix Absorbance Coordinates (WAMACs) are spectral ranges, where specific water absorbance bands related to specific water molecule conformations (water species and water molecular structures) are found with the highest probability. For the first overtone of water (1,300–1,600 nm), 12 WAMACs have been experimentally discovered and confirmed by overtone calculation. The changes in water absorbance spectral pattern (WASP) can be seen in the aquagram (Muncan and Tsenkova, 2019). NIR spectroscopy and Aquaphotomics are used to diagnose Paratuberculosis in dairy cattle by blood plasma (Behdad et al., 2024b) and inflammatory bowel disease (IBD) in humans; its types include Crohn’s disease (CD) and ulcerative colitis (UC) by blood plasma and saliva samples, with high accuracy (Behdad et al., 2024a).

**Figure 1:**
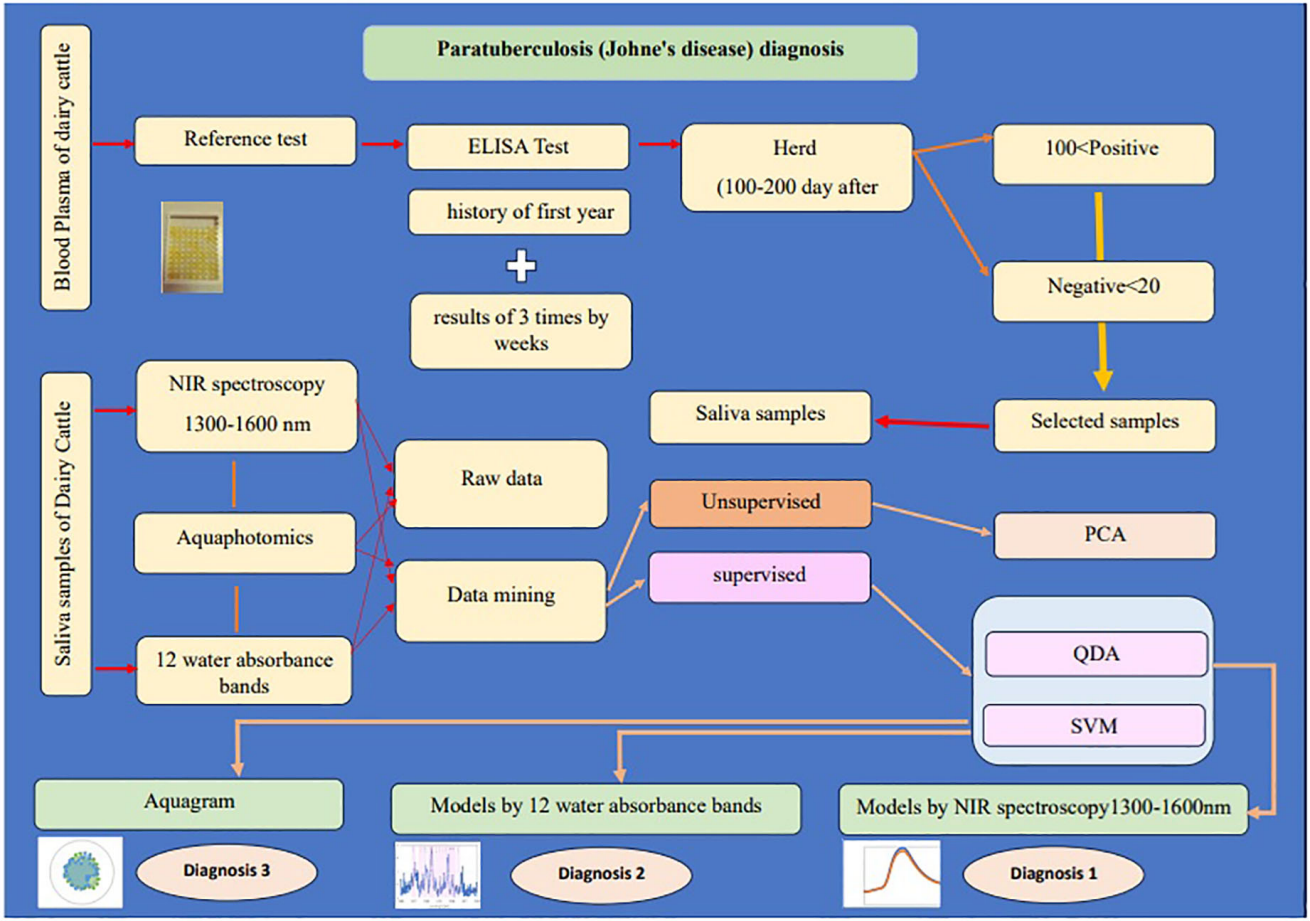
The abstract of the study “Saliva NIR spectroscopy and Aquaphotomics: A novel diagnostic approach to Paratuberculosis in dairy cattle”.

This study aims to use NIR spectroscopy and Aquaphotomics to detect WASP changes in the saliva of dairy cattle for biomonitoring and bio-diagnosis of Paratuberculosis. **Figure 1** displays the abstract of this study.

## 2 Materials and methods

This research was approved by the SRBIAU-Institution of Animal Science (SRB-11-3997) and complied with institutional, national, and international ARRIVE guidelines.

### 2.1 Animals

The study involved using second- and third-lactation stage Holstein dairy cows to test for Johne’s disease. Since the ELISA test for Johne’s disease is not 100% accurate and results can vary at different stages, the test is not able to accurately diagnose the disease in cows in their first year of lactation. Therefore, to obtain the maximum number of samples, cows in their second and third lactation stages were chosen, as they represent the largest population in dairy cow herds.

Out of 150 second- and third-lactation cows, those that consistently scored below an index of 20 in the 1-year-old blood plasma ELISA tests, three weekly ELISA tests before the start of the experiment, and three simultaneous ELISA tests with blood and saliva sampling (totaling seven tests) were classified as healthy, while those with an index of more than 100 were classified as infected animals.

Additionally, the selected cows were free of any other diseases. Only cows with no history of disease since birth, as documented in their health files, were chosen. Furthermore, the veterinarian examined the cows before each sampling day to ensure their health or confirm the absence of any other diseases. Finally, 10 animals were selected for the study (**Table 1**).

**Table 1:** Characteristics of saliva samples from dairy cattle and spectra for the detection of Paratuberculosis in the 1,300–1,600 nm range, including 12 water absorbance bands, over a 3-week period.

Contents	Total number of cows	Number of cows according to the blood plasma ELISA test		Number of spectra	
		Positive	Negative	Positive	Negative
**100–200 days after calving** **(first week)**	10	7	3	21	9
**100–200 days after calving** **(second week)**	10	7	3	21	9
**100–200 days after calving** **(third week)**	10	7	3	21	9
**Total**	10	7	3	63	27

### 2.2 Blood plasma and saliva collection

The animals used in this study were not isolated from the herd. The farm had a routine program for monitoring Paratuberculosis, and the blood samples were taken by the staff for ELISA tests. The blood sample tubes were collected for cows whose ELISA test index of less than 20 and more than 100 was confirmed during 3 weeks (*n* = 50) and then blood samples were taken three times from selected cows that had the same results three times within 3 weeks (*n* = 10). The blood plasma was used for the ELISA test with an IDEXX kit.

The IDEXX kit, as an ELISA kit, has an enzyme immunoassay for the detection of antibodies directed against MAP in bovine individual serum, plasma, and milk samples. First, coated plates were obtained and the sample position was recorded. The Negative Control (NC) was diluted 1:20 in the dilution buffer N.12 and dispensed in one well. The Positive Control (PC) was diluted 1:20 in the dilution buffer N.12 and dispensed in two wells. Plasma samples were diluted 1:20 in the dilution buffer N.12. After homogenizing contents using a microplate shaker, they were incubated for 15 min to 2 h at 18–26°C and then 100 µL was transferred from each well to the preplate to appropriate wells of the coated microplate. Afterwards, the contents of the wells were homogenized by a microplate shaker and were covered with the aluminum cover of the kit and incubated for 45 min at 18–26°C. Then, the solution was removed and each well was washed three to five times with approximately 300 µL of wash solution. Then, 100 µL of conjugate was dispensed into each well, covered, and incubated for 30 min. The solution was removed and each well was washed three to five times with approximately 300 µL of wash solution. Then, 100 µL of TMB substrate N.9 was added into each well and incubated for 10 min at 18–26°C away from direct light. Next, 100 µL of stop solution N.3 was dispensed into each well. The optical density values of samples and controls were measured and recorded at 450 nm. For calculation of controls, Equation 1 is applied, and for plasma samples, Equation 2 is applied (IDEXX Paratuberculosis screening 06-07130-27 manual):


(1) $$\text{Control}:PC\bar{X}\;=\frac{\;PC1\left(450\right)+PC2\left(450\right)}{2}$$

(2) $$\text{Validity criteria}:\frac{PC\bar{X}}{NC}\;A\left(450\right)\geq 3.00\text{ and }PC\bar{X}\geq 0.350$$

Interpretation for plasma samples:


$$\left(\text{Sample}/\text{Control}\right)S/P\%=100*\frac{sample\;A\left(450\right)-NC\;A\left(450\right)}{PC\bar{X}-NC\;A\left(450\right)}$$

Negative:S/P%<45%

Suspect:45%<S/P%<55%

Positive:S/P%≥55%

In this study, the accuracy of the reference test was increased by ensuring that the animals had consistent results of (sample/control) S/P in seven tests, with results ≥100 considered positive and results ≤20 considered negative. Ten animals meeting these criteria were selected for the saliva study.

Saliva samples were collected under the supervision of the farm veterinarian by attaching a sterile nylon to the animal’s lower jaw after restraining its neck. The collected saliva was filtered to remove any food particles and stored in two 30-mL sterile containers. The first container was sent to the laboratory for ELISA and PCR analysis. From the second container, six 2-mL microtubes were prepared and stored in a −23°C freezer for spectrometry. Three of the microtubes were used for the spectrometry.

The samples were transferred to the spectroscopy on ice and warmed for 1 min before spectrophotometry. For each cuvette spectrophotometry, samples were taken from one of the microtubes, and three replicates were taken from each cuvette.

### 2.3 Reference test

In this study, to evaluate the accuracy of NIR spectroscopy and Aquaphotomics models, a reference test method was used. The reference test involved using identical blood ELISA test results from the first year of birth, three consecutive weeks before the test, and three consecutive weeks during the test. Animals with an index of less than 20 were considered healthy, while those with an index greater than 100 were considered infected. Since the ELISA test is used for blood and milk samples, saliva samples were sent for PCR testing. However, the PCR test was unable to differentiate between these samples. As a result, seven identical blood ELISA test results were selected as the reference test.

### 2.4 NIR spectral signature collection

NIR absorbance spectra of saliva were measured using a spectrophotometer (UV-VIS-NIR 3600, Shimadzu CO. Japan) equipped with a quartz cuvette having a 1-mm optical path length (*n* = 90). The samples were defrosted over ice for 15 min and then warmed between the hands for about a minute before collecting NIR spectra. The NIR spectrum was collected in the 1,280–1,630 nm range (interval = 0.5 nm; single scan; very slow). The 1,300–1,600 nm range was used for monitoring and model development. Before collecting saliva spectra, a reference spectrum was taken from two empty cuvettes and then from one empty cuvette and a cuvette containing distilled water. Three independent spectral signatures were collected per sample by replacing the cuvette with saliva between each replicate.

### 2.5 Multivariate analysis

Chemometrics-based multivariate analysis (MVA) was performed using Unscrambler X v.10.5 on the first overtone region of the NIR spectrum, specifically on the vibrational combination band between 1,300 and 1,600 nm. The dataset was pre-treated using various mathematical techniques, such as linear baseline correction, standard normal variate (SNV) with detrending (polynomial order and a first derivative; symmetric Savitzky–Golay smoothing, points = 12), smoothing, normalize, multiplicative scatter correction (MSC), and spectroscopic (absorbance to transmittance). This preprocessing was applied to all databases, including saliva spectra from 10 cattle. A balanced dataset was created by spectral signatures for each category, which were healthy or negative and infected or positive (**Table 2**). Principal component analysis (PCA) was used to reduce the dimensionality of the dataset and identify patterns in spectral behavior. This was achieved by ignoring data labels and detecting excluded data (Ghasemi et al., 2013). A calibration set was created for discriminant analysis, which involved quadratic discriminant analysis (QDA) and support vector machine (SVM) as supervised methods. QDA is a qualitative classification method that can classify new and unknown samples based on models made separately for each group. It helps interpret the differences between groups when the variability of each group does not have the same structure (unequal covariance matrix) and the shape of the curve separating the groups is not linear (Hastie et al., 2009). The QDA method was used to describe the non-linear relationship between groups in the raw data and transformed spectra in 1,300–1,600 nm and 12 water absorbance bands separately. SVM is a supervised learning algorithm that helps find an optimal hyperplane or classifier to classify objects of different classes as accurately as possible. The algorithm tries to maximize the distance between the hyperplane and the points of both groups while leaving the largest possible fraction of points on the same side. This ensures that the algorithm is not overfitting the data and is effectively avoiding the problem of misclassification on the training set. Because of these advantages, SVM is widely used in binary classification problems (Ghasemi et al., 2013). The SVM analysis was used to classify two groups of saliva samples, positive and negative for Paratuberculosis. After that, Aquaphotomics analyses were performed, which included repeating all the above steps only for 12 water absorbance bands in the range of 1,300–1,600 nm. Then, the changes of water bands in infected and healthy saliva samples were drawn as an aquagram. To test mathematical preprocessing and modeling bias against the null hypothesis (no biological signature can differentiate between saliva samples from two classes) in the supervised analysis, datasets were created by positive and negative groups. The samples were randomly divided into a calibration subset for the internal validation set (75%) and a test subset used as the external validation set (25%). This calibration equation was obtained from different sample sets: all samples according to the results of the blood plasma ELISA test as a reference test include a calibration set of positive (>100) and a calibration set of negative samples (<20).

**Table 2:** Results of saliva samples from dairy cattle for the detection of Paratuberculosis in the 1,300–1,600 nm range and 12 water absorbance bands using QDA and SVM models with raw data and pretreatments.

Model	Area	Contents	Predicted model		
			Full data—full validation	Calibration—full validation	Test—cross-validation
**QDA**	**1,300–1,600 nm**	**Negative**	100% (25/25)	100% (19/19)	67% (4/6)
		**Positive**	98% (62/63)	98% (48/49)	100% (14/14)
		**Total accuracy**	99%	98.5%	90%
		**Pretreatment**	Raw data—all pretreatments	Raw data—all pretreatments	Smoothed
		**PC**	17	15	15
	**12 water absorbance bands**	**Negative**	100% (25/25)	100% (19/19)	67% (4/6)
		**Positive**	98% (62/63)	98% (48/49)	100% (14/14)
		**Total accuracy**	99%	98%	90%
		**Pretreatment**	Raw data—all pretreatments	Raw data—all pretreatments (except smoothed)	MSC—smoothed
		**PC**	16-18	13-15	14
**SVM**	**1,300–1,600 nm**	**Negative**	100% (25/25)	100% (19/19)	83% (5/6)
		**Positive**	98% (62/63)	98% (48/49)	93% (13/14)
		**Total accuracy**	99%	98.5%	90%
		**Pretreatment**	All pretreatment	Raw data—normalized	Raw data
		***R* ^2^–RMSEC**	95%–0.1	96%–0.1	
	**12 water absorbance bands**	**Negative**	100% (25/25)	100% (19/19)	83% (5/6)
		**Positive**	98% (62/63)	98% (48/49)	93% (13/14)
		**Total accuracy**	99%	98.5%	90%
		**Pretreatment**	Raw data—smoothed—spectroscopic	Raw data—smoothed—spectroscopic	Raw data—smoothed—spectroscopic
		***R* ^2^–RMSEC**	95%–0.1	96%–0.1	

PC, number of principal components; *R* ^2^, coefficient of determination and RMSEC, root mean square error of calibration.

### 2.6 Evaluation of classification methods

To assess the performance of a classification method, certain quality parameters such as accuracy, sensitivity, and specificity are used. Sensitivity measures the model’s ability to correctly identify true positives of a particular disease, as described by the formula: TP (True Positive)/(TP + FN) (False Negative). It is important to have a high sensitivity (>90%) when using prediction models to identify severe but treatable diseases. On the other hand, specificity measures the model’s ability to correctly identify uninfected or healthy samples. True negative is calculated using the formula: TN (True Negative)/(TN + FP) (False Positive), and the total accuracy is shown by Equation 5.


(3) Sensitivity%=(TP/TP + FN) *100

(4) Specificity%=(TN/TN+ FP)*100

(5) $$\begin{array}{c} \text{Total accuracy}\%\\ \;=\;(((\text{TN}*(\text{TN}/\text{TN }+\text{ FP}))\;+\;(\text{TP}*(\text{TP}/\text{TP}\\ \;+\text{ FN})))/\;(\text{TN }+\text{ FP }+\text{ TP }+\text{ FN}))*100 \end{array}$$

### 2.7 Aquaphotomics

In this research, the contribution of water absorbance bands was investigated to the detection of Positive and Negative saliva groups in Paratuberculosis separately. Therefore, two stages of analyses are all about wavelengths of saliva samples in the range of 1,300–1,600 nm (first overtone of water) and then the wavelength of only 12 water absorbance bands in this range was characterized as follows:

C_1_, 1,336–1,348 (2ν_3_:H_2_O asymmetric stretching vibration); C_2_, 1,360–1,366 [OH-·(H_2_O)_1,2,4_: water solvation shell], C_3_, 1,370–1,376 (ν_1_ +ν_3_: H_2_O symmetrical stretching vibration and H_2_O, asymmetric stretching vibration); C_4_, 1,380–1,388 [OH-·(H_2_O) 1,4: water solvation shell, O_2_-·(H_2_O) 4: hydrated superoxide clusters, 2ν_1_: H_2_O symmetrical stretching vibration]; C_5_, 1,398–1,418 [water confined in a local field of ions (trapped water), S_0_: free water, water with free OH-]; C_6_, 1,421–1,430 (water hydration band, H-OH bend and O-H … O); C_7_, 1,432–1,444 (S_1_: water molecules with one hydrogen bond); C_8_, 1,448–1,454 [OH-·(H_2_O)_4,5_: water solvation shell]; C_9_, 1,458–1,468 (S_2_: water molecules with two hydrogen bonds, 2ν_2_ +ν_3_: H_2_O bending and asymmetrical stretching vibration); C_10_, 1,472–1,482 (S_3_: water molecules with three hydrogen bonds); C_11_, 1,482–1,495 (S_4_: water molecules with four hydrogen bonds); C_12_, 1,506–1,516 (ν_1_: H_2_O symmetrical stretching vibration, ν_2_: H_2_O bending vibration, strongly bound water) (Munćan et al., 2019).

Finally, the results of the study on water absorbance bands are shown by the aquagram according to Equation 6


(6) A'λ=(Aλ−µλ)/σλ

where *A*’*λ* is the normalized absorbance value displayed on the radar axis; *Aλ* is absorbance after scatter correction (multiplicative scatter correction using the mean of the dataset as a reference spectrum or standard normal variant transformation); µ*λ* is the mean of all spectral; *σλ* is the standard deviation of all spectral; and *λ* are the selected wavelengths from WAMACS regions corresponding to the activated water absorbance bands (Munćan et al., 2019).

## 3 Results

### 3.1 Raw absorbance spectra of saliva

The bovine saliva contained up to 99% water and 1% inorganic (sodium, potassium, phosphates, chloride, calcium, and magnesium) and organic components, which are divided into two: proteins and non-proteins (Carpenter, 2013).

According to the characteristics of the spectra obtained in the NIR range, machine learning methods called chemometrics were used to obtain hidden information (Tsenkova et al., 2018).

One of the main data preparation operations is data cleaning, which includes smoothing noises, identifying and removing outliers, and resolving inconsistencies. In this research, no noise was observed in the saliva spectra. The studied range was determined to be 1,300–1,600 nm, and to be sure, the spectra were taken from the range of 1,280 to 1,630 nm.

The raw NIR absorbance spectra of saliva samples in the analyzed range 1,300–1,600 nm are presented in **Figure 1**. These spectra seem identical in this spectral region, whose main feature is a dominant absorbance band approximately 1,450 nm attributed to the first overtone of OH stretching vibration (Vitalis et al., 2023). Since bovine saliva is made up of 99% water, the spectra of saliva are similar to the water spectra.

**Figure 2:**
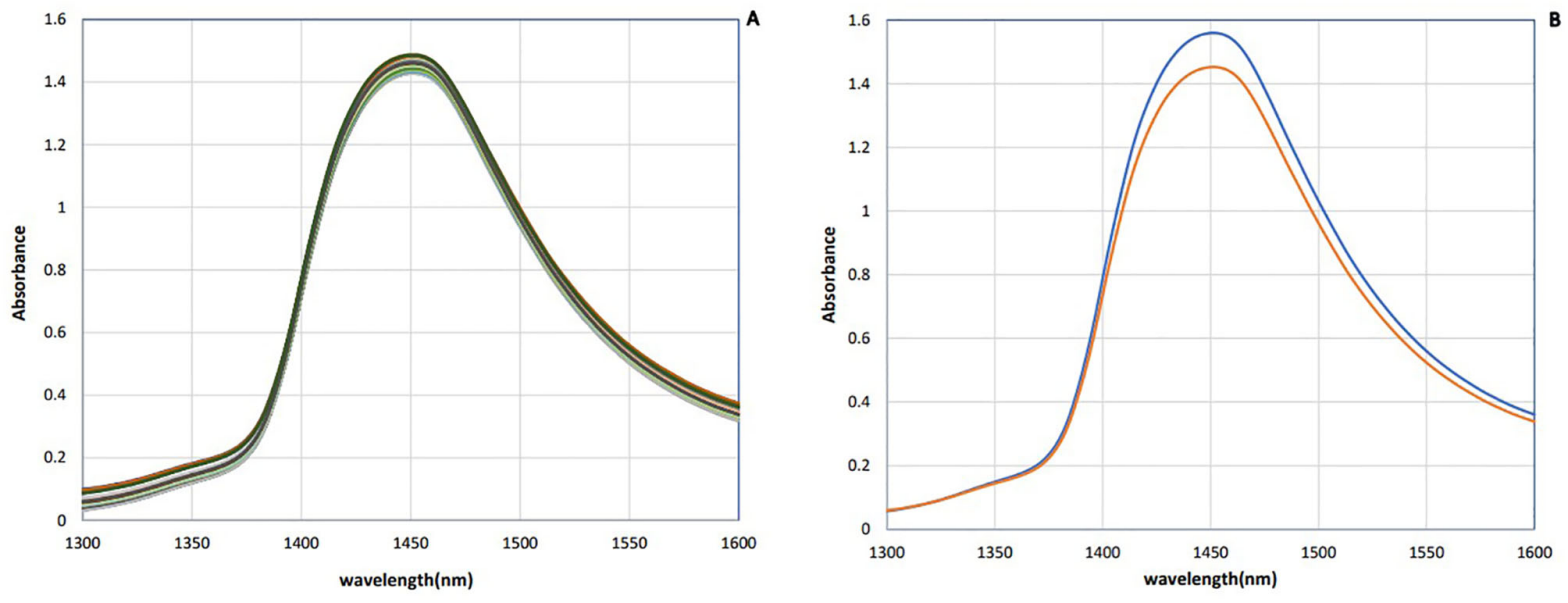
Saliva absorbance NIR spectra of dairy cattle: **(A)** Raw data. **(B)** Mean of data (*n* = 90). Negative (blue) and positive (red).

**Figure 3:**
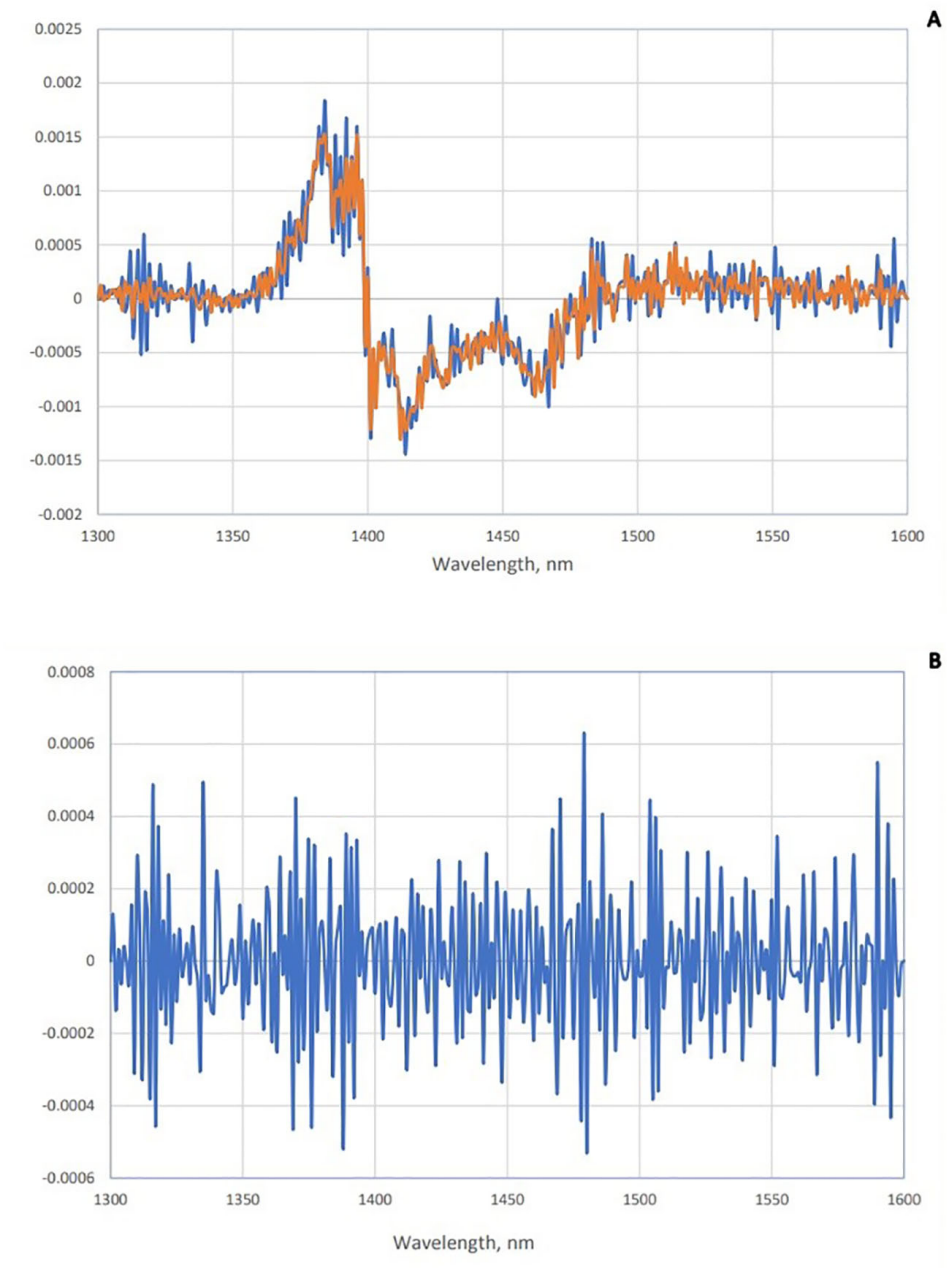
Subtraction spectra of positive and negative groups of saliva. **(A)** Subtraction spectra of second derivative data from positive (red) and negative (blue) groups of saliva from the total mean spectra in Paratuberculosis. **(B)** Subtraction spectra of second derivative data of positive and negative groups of saliva from each other in paratuberculosis.

To enhance the subtle changes at specific water absorbance bands in the spectra of samples, means and spectral subtraction were performed in the initial evaluation (Tsenkova et al., 2018). The mean absorption spectrum of saliva in the raw data was consistent with the pattern of the original data, and at 1,450 nm, the highest absorption rate of both groups was observed (**Figure 2A**). The mean of absorption for the negative group was higher than the positive group, and this difference was evident in **Figure 2B**. To enhance the subtle differences, the average spectra of the second derivation of the positive and negative groups were subtracted from the total average of all spectra (**Figure 3A**). Another way to enhance the differences is to calculate the difference spectrum between the average spectra of the second derivation of positive and negative groups (**Figure 3B**). This spectral subtraction enhanced the differences between the two groups in the second derivation spectral subtraction; all 12 water absorbance bands are active and the differences are so obvious.

### 3.2 Principal Component Analysis–exploratory analysis of Paratuberculosis effects on spectra of saliva

**Figure 4:**
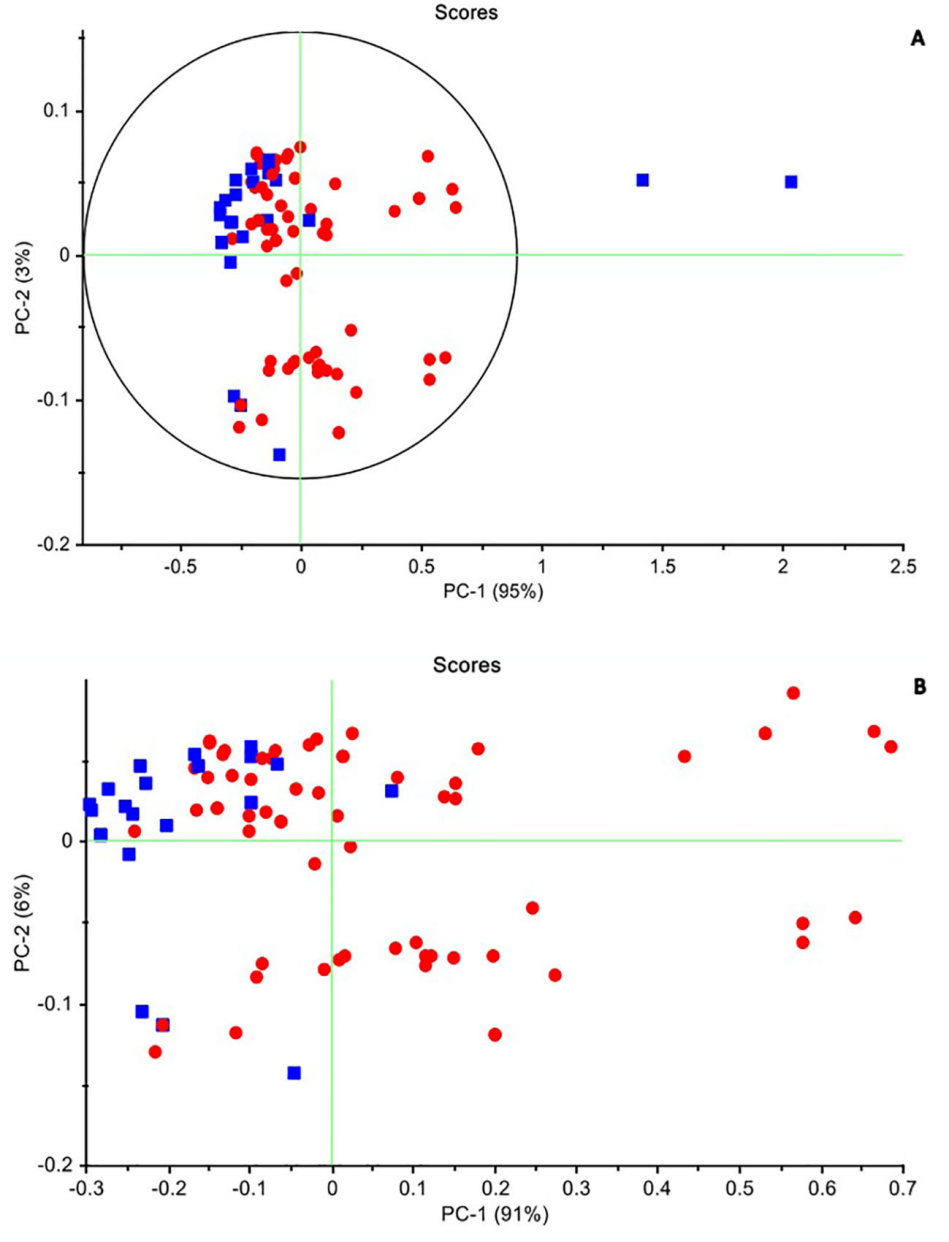
The principal components analysis (PCA) method in two healthy and infected groups. **(A)** Raw data. PC1: 95% and PC2: 3%. The samples that were outside the Hotling T^2^ ellipse with an accuracy of 95% were recognized as outliers and were removed from the calculated data category. **(B)** The smoothed data after excluded the two outline samples. PC1: 91% and PC2: 6%, which cover 97% of the differences between the two groups. Negative (blue) and positive (red).

The PCA is an unsupervised multivariate analysis that reduces the dimensionality of datasets, explains the variation in the data by ignoring the data label, helps in the detection of patterns in the spectral behavior, and finds excluded data (Ghasemi et al., 2013). The results of PCA were presented as scores and loading plots. In the PCA score plot, the similarity and differences in chemical complexes that contain OH, CH, and NH bonds interact with NIR light in transformed spectra of bovine saliva in 1,300–1,600 nm range can be observed in **Figure 4**. Based on this, by performing PCA on all data, in accordance with the established criteria, the samples that were outside the Hotling T^2^ ellipse with an accuracy of 95% were recognized as outliers and were removed from the calculated data category. From the total of 90 independent spectra obtained from both healthy and diseased classes, two spectra related to one animal (2.2% of all samples) were recognized as outliers and removed from the dataset. Subsequent analyses were performed on the remaining 88 samples.

According to the scores chart of the raw data, the first three principal components describe all the variance of the total data, so that PC1 is 95% and the share of the other two PCs is 3% and 1%, respectively. **Figure 4A** shows the distribution of samples in both healthy and infected classes of raw data obtained in the PC1–PC2 space. As can be seen, despite some mixing between healthy and infected samples in the PC1–PC2 space, these two groups were separated from each other to the acceptable extent expected from a non-learning analysis such as PCA. While the healthy samples tended to be placed in the second quadrant of the PC1–PC2 space, the infected samples were more scattered in the first to fourth quadrants of this space. According to the descriptive variance by the first two components and the separation algorithm, it is expected that the discernment analysis such as QDA can better separate these two groups of samples. PCA was performed also with various pre-processing techniques. In the normalized pre-processing, PC1 and PC2 describe 93% and 7% of the variance of the total data, respectively. The healthy samples were located in the second and third quadrants, while the infected samples were mostly scattered in the first, second, and third quadrants. After applying smoothing to the data, the separation improved, with healthy samples clustered in the second quadrant. PC1 and PC2 then described 91% and 6% of the total data variance, respectively (**Figure 4B**).

### 3.3 Quadratic Discrimination Analysis for the detection of dairy cattle response to Paratuberculosis of saliva

QDA as a discriminant analysis, when the variability of each group does not have the same structure (unequal covariance matrix) and the curve shape of separating groups is not linear, will provide a better classification model (Hastie et al., 2009).

QDA in the range of 1,300–1,600 nm had sensitivity and specificity in internal validation for the total wavelength of raw data, and all pretreatments were 100% and 96%; only 1 sample out of 63 infected samples was misdiagnosed, and for calibration, data were 100% and 95%, respectively, with only one misdiagnosis. In external validation, the positive and negative groups were separated by 100% sensitivity, 67% specificity, and 90% accuracy in smoothing pretreatment (**Table 2**).

QDA in WAMACs in saliva in internal validation has 100% and 100% sensitivity and 96% and 95% specificity for full data and calibration, respectively. In external validation, smoothed or MSC pretreatment accrued to 90% total accuracy, 100% sensitivity, and 67% specificity in Paratuberculosis detection. The similar results of two ranges, 1,300–1,600 nm and 12 water absorbance bands, show that the water bonds had a main role in the detection of healthy and infected animals by saliva samples.

### 3.4 Support Vector Machine analysis for the detection of cattle response to Johne’s disease of saliva

SVM has been used to detect healthy and infected groups as a powerful supervised method. According to SVM analysis, the prediction equations obtained from the raw data and all pretreatment models, which were the result of internal validation of full data and calibration, contained 99% and 100% total accuracy, 100% specificity with 98% and 100% sensitivity, and the total accuracy, sensitivity, and specificity for external validation were 90%, 93%, and 83%, respectively. In the separation of negative and positive groups, these models yielded a high coefficient of determination (*R*²) of 94% and 100%, and low root mean square error of calibration (RMSEC) of 0.11% and 0.05% (**Table 2**).

This time, SVM was performed based on Aquaphotomics. In comparison to the data obtained from SVM by WAMACs with the results obtained from all wavelengths in the range of 1,300–1,600 nm, in internal validation models with raw data and smoothed or spectroscopic (absorbance to transmittance) pretreatments, full data and calibration had 99% total accuracy, 98% sensitivity, and 100% specificity. The coefficient of determination, *R*² = 94% and 95%, and RMSEC = 0.1 were obtained in separating the negative and positive groups.

External validation shows that in the model with raw data or smoothed or spectroscopic (absorbance to transmittance) pretreatment, total accuracy in separating positive and negative groups was 90%, sensitivity was 93%, and specificity was 83%.

Internal and external validations show that in the models with raw data, smoothed and spectroscopic pretreatments achieved the same total accuracy of 90% in separating positive and negative groups for Paratuberculosis, demonstrating the high contribution of water bands in the results of the SVM models in the 1,300–1,600 nm range.

These findings demonstrate that by observing the spectral profile of dairy cow saliva in the 1,300–1,600 nm range using NIR spectroscopy, Aquaphotomics, and data mining, it is possible to detect changes in functional groups and active water absorbance bands in response to the perturbation—Johne’s disease. This method accurately diagnosed and classified healthy and infected groups that were selected according to the results of seven blood plasma ELISA tests (**Table 2**).

### 3.5 Aquagrams

**Figure 5:**
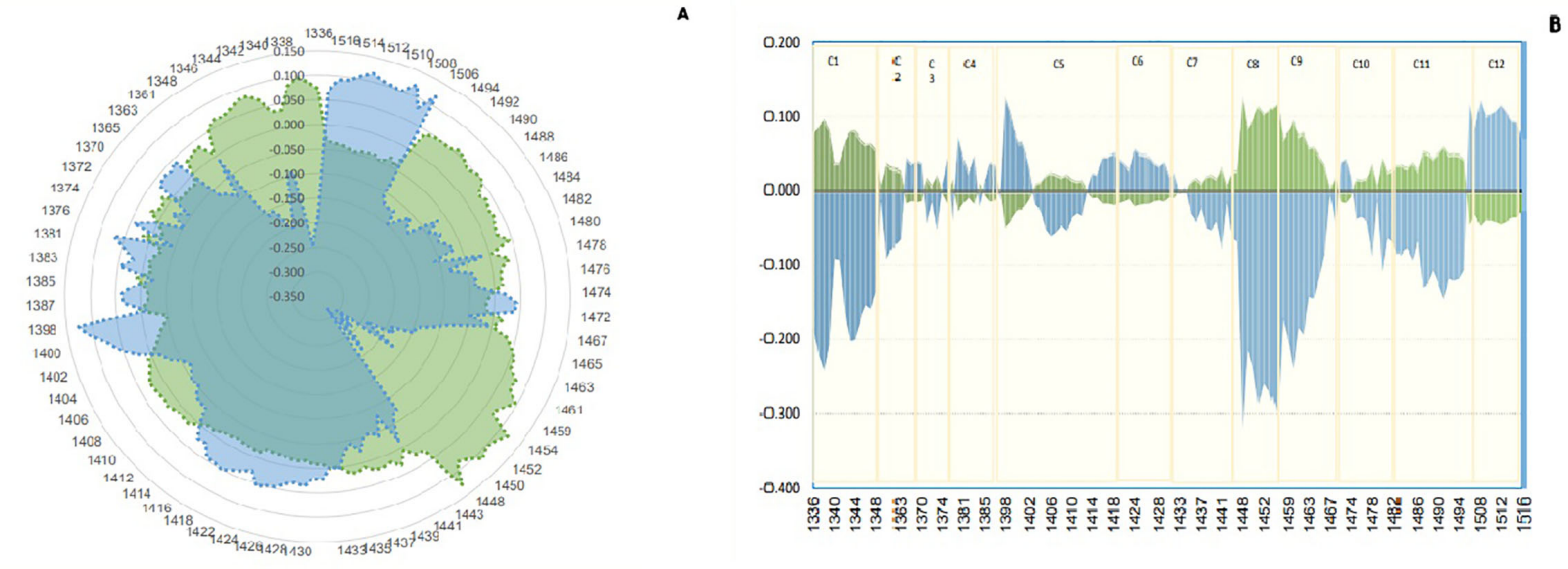
Aquagram. The changes in 12 water absorbance bands of dairy cattle saliva - normalized using multiplicative scatter correction (MSC)- visualized the differences in Water Absorbance Spectral Patterns (WASPs) between positive (green) and negative (blue) groups of Paratuberculosis. **(A)** Radar aquagram. **(B)** Linear aquagram. The axes represent the 12 water absorbance bands, marked in yellow.

The study aimed to determine the difference in water structure between the saliva of individuals infected with Johne’s disease and those who are not. The aquagram, which displays normalized absorbance values from MSC pretreatment at water bonds, was used to investigate the water absorbance bands that responded strongly to Johne’s disease. By comparing the aquagram for the positive and negative groups, the relationship between healthy and infected individuals with WASP was estimated. Based on the results of the mean spectrum difference, all WAMACs wavelengths distinguish between positive and negative groups and are thus used in drawing the aquagram. According to the diagram, active water bands include C_2_: water solvent shell, C_3_: water bands with symmetric and asymmetric stretching vibration, C_4_: hydrated superoxide clusters and symmetrical stretching vibration, C_5_: containing free water, C_6_: free OH bands and hydrated water, and C_7_: bands with a hydrogen bond were same approximately. However, in C_1_: water with asymmetric stretching vibration, C_8_: water solvation shell, C_9_: water molecules with two hydrogen bonds, C_10_ with three hydrogen bonds, and C_11_ with four hydrogen bonds, positives are increased, but C_12_ with stronger bonds decreased. In other words, the effect of changes in antibody blood plasma causing Johne’s disease can be observed in WASPs of saliva, where the free water proportion, hydrated, and having a hydrogen bond in the saliva of negative and positive are about the same, but asymmetric stretching vibration bond and water molecules with two, three, and four hydrogen bonds are increased in positive samples, and water that is strongly bound and with symmetrical stretching vibration is significantly reduced. Moreover, as the amount of antibodies in Johne’s disease in blood plasma changed, the proportion of active water bonds or WAMACs in saliva was also changed (**Figure 5**).

## 4 Discussion

Saliva is a biofluid that, like a mirror, reflects the state of body health or perturbation. Vibrational spectroscopy, Raman, and infrared can provide a detailed salivary fingerprint that can be used to discover biomarkers for disease diagnosis (Derruau et al., 2020). A recent study investigated the use of NIR spectroscopy and Aquaphotomics to diagnose Paratuberculosis in dairy cattle 100–200 days after calving, using saliva samples. Separate datasets were created for the positive and negative groups, and the study tested the null hypothesis that no biological signature can distinguish between saliva samples from these two groups. The current study used NIR spectroscopy and Aquaphotomics to perform unsupervised and supervised analysis in two ranges, 1,300–1,600 nm and only in 12 water absorbance bands in this range. In other words, this study aimed to investigate the ability of biomonitoring and diagnosis of NIR spectroscopy and Aquaphotomics in detecting spectral changes and 12 water absorbance bands of saliva due to Paratuberculosis. Data mining for the study was done based on multivariate classification analysis. The initial classification of two healthy and infected groups for supervised modeling was done based on the results of seven ELISA tests, in the first year of birth, and six consecutive weekly tests, as a reference method.

NIR spectroscopy is a useful tool for analyzing raw materials like milk, particularly on portable instruments that can be used directly on the dairy farm (Takemura et al., 2015). It can also be used to detect genetically modified organisms, and Aquaphotomics is a novel field that has the potential to monitor them (Sohn et al., 2021). By profiling the physiological and metabolic changes in dairy cattle, NIR spectroscopy can distinguish between healthy and infected animals, and it can be used to detect and monitor infections like Johne’s disease. Multivariate analysis and the Aquaphotomics approach can be used to examine the biochemical changes that cause differences between the blood plasma of healthy and infected animals, and the profile of NIR spectra can reflect the animal’s immune response to the disease agent (Meilina et al., 2009; Morita et al., 2013; Ramirez-Morales et al., 2021; Santos-Rivera et al., 2021).

Infrared and NIR spectroscopy were even used in 2012 to detect *Mycobacterium tuberculosis*. In 2024, NIR spectroscopy and Aquaphotomics were used to diagnose Paratuberculosis in dairy cattle by blood plasma sample (Behdad et al., 2024b) and IBD in humans by blood plasma and saliva sample (Behdad et al., 2024a) with 100% accuracy, which show the strong ability of NIR spectroscopy and Aquaphotomics in the diagnosis of disease.

This study used PCA, QDA, and SVM to analyze saliva spectra, which provide information about biochemical imbalances resulting from diseases that affect the composition of saliva. These changes in saliva’s structure affect the water present in saliva, characterized by the reuse of PCA, QDA, and SVM in the range of 12 bands of water that can be viewed in the aquagram. The raw data spectrum and the average of negative and positive groups confirmed the high proportion of water in saliva compounds, with a peak at 1,450 nm. The PCA, in raw data, separated the positive and negative groups as expected from the unsupervised method, with the positive and negative classes on the right and left of the graph, respectively. By normalized pretreatment, the first two PC loadings explained 93% and 7% of the dataset variance, respectively. Previous studies have used PCA methods to diagnose mastitis in dairy cattle by identifying NIR features of milk, blood plasma, and urine (Tsenkova et al., 2001; Morita et al., 2013; Ramirez-Morales et al., 2021; Muncan et al., 2022).

In the QDA model, the results of full data and 12 water absorbance bands were the same in internal and external validation. In the internal validation, the raw data in the full range and all pretreatments achieved 99% and 95.5% total accuracy, 100% and 100% sensitivity, and 96% and 95% specificity, respectively. In addition, in external validation, the total accuracy, sensitivity, and specificity were 90%, 100%, and 67%, respectively. These results demonstrate the decisive role of water in separating the two groups.

The prediction equation was obtained from the raw data and pretreating models in internal full data and calibration, and external validation for the SVM analysis. This model provided 100% specificity with 98% and 100% sensitivity in the internal validation of full data and calibration. The external validation resulted in a total accuracy of 90%, a sensitivity of 93%, and a specificity of 83%. In separating negative and positive groups, these models have a high coefficient of determination (*R* ^2^) of 99.9% and a low error coefficient (RMSEC) of 0.05%. Following that, the SVM models were used in internal validation models of full data in water bands (WAMACs) with the same results in the 1,300–1,600 nm range, in the separation of negative and positive groups. In the internal validation of the calibration model, 100% specificity and 98% sensitivity were achieved. External validation shows the same results to 1,300–1,600 nm in separating positive and negative groups for Paratuberculosis. This result indicates a high contribution of water bands of saliva in the results of the SVM models in the 1,300–1,600 nm range. The combination of NIR spectroscopy with aquaphotomics is effective for developing an accurate and rapid early diabetes diagnosis model. In this study, according to the SVM model, accuracy was 97.22%, and specificity and sensitivity were 95.65% and 100%, respectively, in the first-derivative pretreatment (Li et al., 2020).

It seems that, because of the amount of water in saliva, the changes and response to the disturbances resulting from Johne’s disease are evident and all the factors that separate the two groups are summarized in the changes of water bands. The same results of the developed models in a total range of 1,300–1,600 nm and only 12 water absorbance bands show the critical role of water bonds in diagnosing Johne’s disease according to the water comprehensive mirror and biomarker approach in aquaphotomics. The 12 water absorbance bands reduce the dimensionality of datasets and increase the accuracy of developed models.

NIR spectroscopy and artificial neural network (ANN), with fecal culture and serum test ELISA as a reference test, were used to diagnose Paratuberculosis in dairy cattle, but it is unclear which parameters or substances in the serum the NIR spectroscopy discriminates (Norby et al., 2006).

In the current study, the total accuracy of using NIR spectroscopy to diagnose Johne’s disease in dairy cattle with a 95% confidence margin is 99%, 100%, and 90% in full data, calibration, and test, respectively. The method’s sensitivity was 100%, which is comparable to the sensitivity of the current diagnostic models, such as the ELISA test (7%–94%), fecal culture (20%–74%), and PCR (4%–100%). The best model uses saliva spectra from QDA in the 1,300–1,600 nm range or only WAMACS.

Water, as a comprehensive biomolecule, strengthens all small and unobservable changes in this range and makes it measurable. In addition, the changes of water absorbance bands in response to the disturbances resulting from Johne’s disease in the body provide a rich layer of information to researchers to enable the diagnosis of Johne’s disease as an accurate and rapid biomarker, along with other available diagnostic methods.

The study had several limitations. Firstly, livestock farms had a routine ELISA testing program for Johne’s disease and a history of testing for all livestock. Secondly, there were issues with inconsistent test results, which led to repeated testing (seven times) and the exclusion of many animals with fluctuating positive results. Thirdly, the test accuracy was very low in the first year, and although its accuracy improved in the fourth and fifth years, the total number of animals in the herd at this age was lower.

The researchers are encouraged to validate the method outlined in this study by using alternative common diagnostic methods, such as fecal culture or PCR, in addition to the ELISA test as a reference test. This validation should be performed on a larger sample size of animals. The aquagram obtained should be documented for each reference test method, breed, and physiological stage of the animal. This documentation is important for the global registration of an aquaphotome—the collection of aquagrams in different conditions—for Johne’s disease.

Facilities such as aquaphotomics and advanced diagnostic equipment such as NIR spectroscopy improve the accuracy and quality of diagnosis diseases such as Johne’s disease in cattle. These facilities assist breeders in detecting diseases in the early stages and providing appropriate treatment by using saliva as a suitable and non-invasive sample. These methods are used for the diagnosis of Johne’s disease in dairy cattle by blood sample (Behdad et al., 2024b) and IBD in humans by blood plasma and saliva samples (Behdad et al., 2024a) with high accuracy. The results of the current study, by using a saliva sample as a non-invasive and simple sample, agree with those findings.

## 5 Conclusion

The study separated dairy cows into two groups based on their results of the first year of birth and six consecutive weekly ELISA blood plasma tests. The healthy group had an index of less than 20, while the infected group had an index of greater than 100. The researchers developed three classification models that used the blood plasma index and the spectral changes in saliva caused by the disease, focusing specifically on the structure of the water absorbance bands of saliva. These models were able to diagnose Paratuberculosis and distinguish between the healthy and infected groups with a total accuracy of 99%, a sensitivity of 98%, a specificity of 100% in internal validation, and a total accuracy of 90% in external validation. The same results of developed models in a total range of 1,300–1,600 nm and only 12 water absorbance bands show the critical role of water bonds in diagnosing diseases according to the water comprehensive mirror and biomarker approach in aquaphotomics. The 12 water absorbance bands reduce the dimensionality of datasets and increase the accuracy of developed models. Additionally, the researchers obtained an aquagram showing the changes in the 12 water absorbance bands of saliva in healthy and infected groups.

The results of this study suggest that NIR spectroscopy and the aquaphotomics approach can accurately diagnose Paratuberculosis in dairy cattle using saliva samples in a non-invasive and timely manner. The researchers recommend conducting a global study on a higher number of animals and using other reference tests to validate this method. They also suggest studying the four stages of paratuberculosis separately, as this method could become the standard for diagnosing paratuberculosis.

## Data Availability

The data underlying this article will be shared on reasonable request to the corresponding author.
